# Using relative and absolute measures for monitoring health inequalities: experiences from cross-national analyses on maternal and child health

**DOI:** 10.1186/1475-9276-6-15

**Published:** 2007-10-29

**Authors:** Tanja AJ Houweling, Anton E Kunst, Martijn Huisman, Johan P Mackenbach

**Affiliations:** 1 Department of Public Health, Erasmus MC University Medical Center Rotterdam, Rotterdam, The Netherlands

## Abstract

**Background:**

As reducing socio-economic inequalities in health is an important public health objective, monitoring of these inequalities is an important public health task. The specific inequality measure used can influence the conclusions drawn, and there is no consensus on which measure is most meaningful. The key issue raising most debate is whether to use relative or absolute inequality measures. Our paper aims to inform this debate and develop recommendations for monitoring health inequalities on the basis of empirical analyses for a broad range of developing countries.

**Methods:**

Wealth-group specific data on under-5 mortality, immunisation coverage, antenatal and delivery care for 43 countries were obtained from the Demographic and Health Surveys. These data were used to describe the association between the overall level of these outcomes on the one hand, and relative and absolute poor-rich inequalities in these outcomes on the other.

**Results:**

We demonstrate that the values that the absolute and relative inequality measures can take are bound by mathematical ceilings. Yet, even where these ceilings do not play a role, the magnitude of inequality is correlated with the overall level of the outcome. The observed tendencies are, however, not necessities. There are countries with low mortality levels *and *low relative inequalities. Also absolute inequalities showed variation at most overall levels.

**Conclusion:**

Our study shows that both absolute and relative inequality measures can be meaningful for monitoring inequalities, provided that the overall level of the outcome is taken into account. Suggestions are given on how to do this. In addition, our paper presents data that can be used for benchmarking of inequalities in the field of maternal and child health in low and middle-income countries.

## Introduction

Reducing health inequalities between social groups within countries is an important public health objective. Monitoring of such health inequalities, therefore, is an important public health task. Comparisons are an integral part of monitoring. The aims of such comparisons are to assess whether health inequalities are smaller or larger compared to other countries [1], whether inequalities have increased over time [2], or whether inequalities develop in the direction of predefined goals [3]. Such monitoring is important, both for high-income countries, and for low and middle-income countries.

There is much debate about the inequality measure to be used for monitoring. There is consensus on the *importance of the choice *of the measure, since this may influence the conclusions drawn [4-7]. However, there is less consensus on *which *measure is most meaningful. The key issue that has raised most recent debate is whether to use relative or absolute measures of inequality [5-7]. According to some authors, extreme caution is needed when using relative measures to monitor inequalities. Increasing relative inequalities, it is suggested, are 'nearly inevitable' when the overall level of the outcome (e.g. mortality) falls. Similarly, ratios in the reverse outcome (e.g. survival), would almost necessarily decrease. This would lead to "diametrically opposed interpretations" of patterns of inequality [7]. This latter problem is obviously avoided when using absolute measures of inequality, such as the rate difference [5]. Others, however, warn that using absolute inequality measures "almost inevitably" leads to smaller inequalities when overall levels fall, and that therefore ratio-based measures are more meaningful for monitoring purposes [8].

Our paper aims to inform this debate and develop recommendations for monitoring health inequalities on the basis of empirical analyses of health-related inequalities in a broad range of developing countries. We examine to what extent relative and absolute inequalities on the one hand, and overall levels on the other, are indeed empirically related as suggested by the above mentioned authors. We also assess to what extent any observed associations can be explained by mathematically-defined ceilings to relative and absolute inequality measures.

We examine the above issues by means of a cross-national analysis of 43 low and middle-income countries for one health outcome (under-5 mortality) and three indicators of health care use (full childhood immunization, skilled antenatal care, skilled delivery assistance), using the Demographic and Health Surveys (DHS) dataset. The DHS is the largest survey program in low and middle-income countries with standardized questionnaires containing information on socio-economic characteristics, mortality, and health care use. A cross-sectional analysis of low and middle-income countries is particularly suitable for answering the above questions because of the wide range of overall levels of health-related outcomes across these countries.

## Methods

Data on poor-rich differences in under-5 mortality, full childhood immunization coverage, skilled delivery attendance and antenatal care for 43 low and middle-income countries were obtained from World Bank Country Reports [9]. The Country Reports are based on DHS data [10]. These are nationally representative surveys, for which usually between 5000–10000 women aged 15 – 49 years were interviewed. The data and indicators used have been described elsewhere in more detail [9]. We included those countries for which Country Reports were available at time of analysis.

Household wealth was the socio-economic characteristic used in this study. Wealth has been shown to be an important determinant of mortality and health care use. It is extensively used in the field of health inequalities research, especially in studies on low and middle-income countries. Wealth was measured using an index based on household ownership of assets. The assets were combined into a wealth index using Principal Components derived weights [9,11]. Despite its limitations [12], this index is fairly widely used as measure of economic status in developing countries [11,13]. The total population in each of the countries was categorized accordingly into five, equally large, wealth layers.

First, scatter plots were used to assess the relationship between the overall level of the health-related outcomes and the magnitude of absolute and relative inequalities in these outcomes. The simplest inequality measures were used, i.e. the rate difference (RD) and the rate ratio (RR) between the poorest 40% and richest 40% population group. We calculated the R-square of the best fitting curve through the scatter plots.

Then, we examined to what extent the empirical patterns of the RR and RD could be clarified by mathematically-defined ceilings to the RR and RD. We calculated these ceilings using a hypothetical population of which 50% is poor and 50% is rich. For example, if overall immunisation coverage is 100%, the RR cannot exceed 1, and the RD cannot exceed 0. If overall immunisation coverage is 90%, the maximum value of the RR is 1.25 (i.e. 100% coverage among the rich and 80% among the poor) and is 20 for the RD. For outcomes that never reach 100%, like under-5 mortality, we made an adjustment to calculate realistic ceilings. We assumed a minimum under-5 mortality of 5 per 1000 live births and a maximum of 400/1000.

## Results

### General tendencies

**Figure 1:**
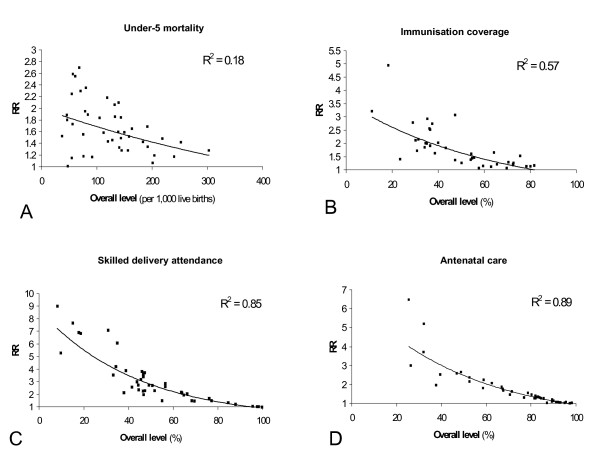
a-d Rate Ratio (comparing the poorest 40% and richest 40% population group) by overall level of the outcome: under-5 mortality, full childhood immunisation coverage, skilled delivery attendance, and skilled antenatal care, for 43 low and middle-income countries. Exponential curves were fitted through the data.

Both the RR and the RD are empirically related with the overall level of the outcomes studied. RRs tend to be higher at lower overall levels, as shown by the trend-lines in Figures 1a–d. The amount of scatter around the trend-line varied between the outcomes. Whereas for skilled delivery attendance and antenatal care the RR was to a high degree (up to 89%) explained by the overall level, the explained variance was quite low for under-5 mortality. For this outcome, mainly the range of the RR was larger at lower overall levels. So, although relative inequalities in under-5 mortality tend to be higher at lower overall levels, even at comparatively low mortality levels there were countries with a low relative inequalities.

**Figure 2:**
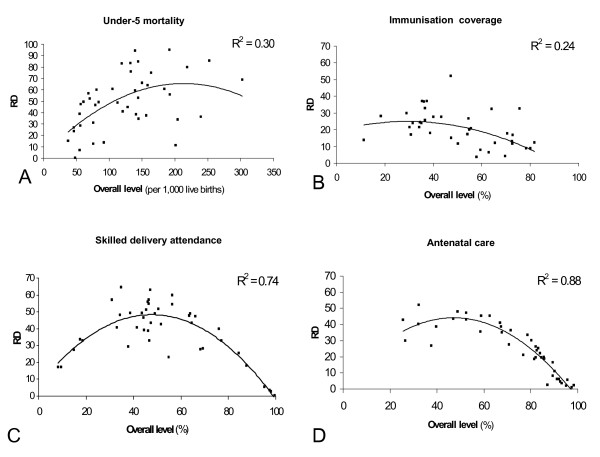
a-d Rate Difference (comparing the poorest 40% and richest 40% population group) by overall level of the outcome: under-5 mortality, full childhood immunisation coverage, skilled delivery attendance, and skilled antenatal care, for 43 low and middle-income countries. Parabolic curves were fitted through the data.

The relationship between the RD and the overall level has the shape of a reverse-u (Figures 2a–d), with low RDs at both high and low overall rates, and high RDs at intermediate levels. The exact pattern, however, varied between the outcomes. For antenatal care and skilled delivery attendance, the pattern approximated a fully reversed-u shape, whereas for other health outcomes, only the left (under-5 mortality), or right (immunisation) part were represented. The extent to which the RD was explained by the overall rate varied between health outcomes, from moderate (R^2 ^= 0.24) to very high (R^2 ^= 0.88). A high R^2 ^implies that there is little variation in the magnitude of the RD between countries with similar overall levels of the outcome.

**Figure 3:**
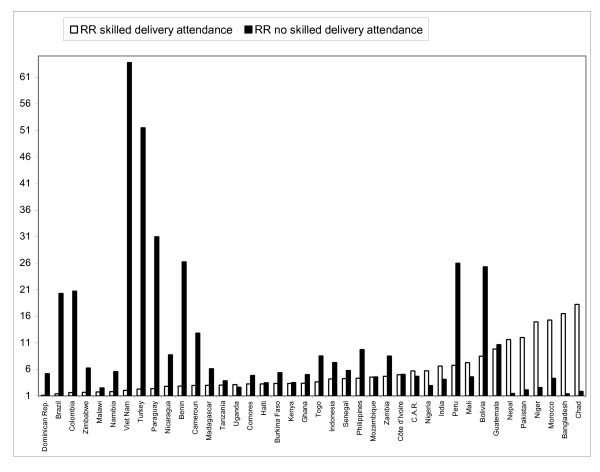
Comparing poor-rich Rate Ratios (richest 20% – poorest 20% population group) in skilled delivery attendance with poor-rich Rate Ratios in prevalence of no skilled delivery attendance.

The magnitude of the RR was sensitive to whether the outcome was defined positively or negatively. In Brazil, for example, poor women were over 20 times more likely not to be attended by a skilled person during delivery than rich women. However, as most deliveries in Brazil were attended by a skilled person, the poor-rich ratio in skilled attendance was only 1.38 (Figure 3). Whereas also the position of countries in terms of the magnitude of the RR was strongly sensitive to whether the outcome was defined positively or negatively, the country rankings were not necessarily diametrically opposed. The correlation coefficient of the ranking of countries was *r *= 0.40 for immunization, 0.32 for under-5 mortality, -0.02 for antenatal care, and -0.47 for delivery attendance. A negative coefficient means that low RRs when using a positive definition of the outcome were associated with high RRs when using a negative definition, and vice versa.

The ranking of countries was for some outcomes, i.e. skilled delivery attendance and under-5 mortality, highly sensitive to whether the RR or the RD was used (rank-correlation coefficient *r *= 0.32 and *r *= 0.39 respectively). For other outcomes, however, the ranking was more robust (immunization coverage: *r *= 0.85, and antenatal care: *r *= 0.97).

### Mathematical ceilings

**Figure 4:**
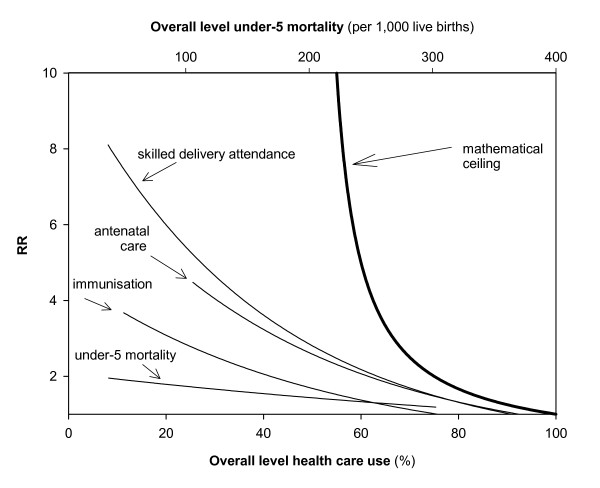
Rate Ratio (comparing poorest 40% and richest 40% population group) by overall level of the outcome, and mathematically defined ceiling to value of RR. The curves presented for the health-related outcomes correspond to those shown in Fig. 1a-d. The upper x-axis gives the overall-level for under-5 mortality. The lower x-axis gives the overall level for immunisation coverage, antenatal care and skilled delivery attendance.

The maximum values of the RR at given overall levels of health care use and under-5 mortality are shown in Figure 4. This mathematically-defined ceiling moves downwards with increasing overall levels. Figure 4 also shows the trend-lines of the RR derived from the empirical observations. The patterns of the observed values resemble the pattern of the mathematically-defined ceiling, with very low RRs at high overall levels. Yet, the ceiling cannot clarify why the RR still tends to increase below overall levels of 50%. For example, the RR tends to be lower at overall levels of 40% than at 10%, even though also at 40% there is no mathematical ceiling.

**Figure 5:**
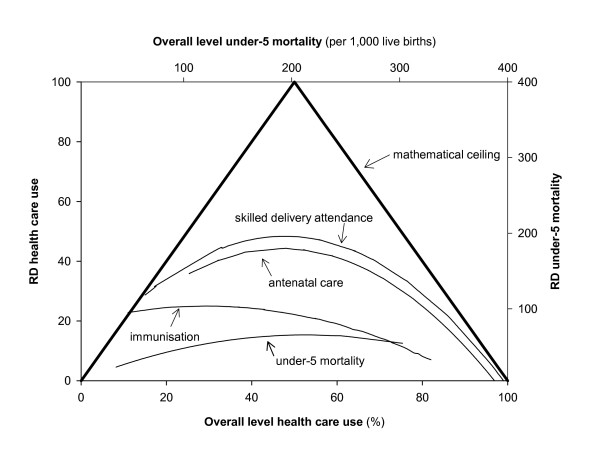
Rate Difference (comparing poorest 40% and richest 40% population group) by overall level of the outcome, and mathematically defined ceiling to value of RD. The curves presented for the health-related outcomes correspond to those shown in Fig. 2a-d. The upper x-axis gives the overall-level for under-5 mortality. The lower x-axis gives the overall level for immunisation coverage, antenatal care and skilled delivery attendance.

The mathematically defined ceiling of the RD is 0 at overall levels of 0% and 100% (Figure 5). From these two points, the ceiling increases linearly, and reaches a maximum of 100 at an overall level of 50%. The empirical trend-lines resemble the pattern of the mathematically-defined ceiling. The strength of this association, however, varied between health outcomes. For delivery attendance and antenatal care, inequalities tended to be rather close to the maximum. Conversely, for immunisation coverage and under-5 mortality, the RDs were systematically lower than the maximum, and the patterns were far from determined by the mathematical ceiling.

The empirically observed low RRs at high overall levels are therefore not surprising. Low RRs at high (>60–70%) overall rates are a *necessity*, not an accomplishment. Therefore, one cannot conclude, for example, that Niger, with a RR of 1.3 is doing comparatively well in terms of relative mortality inequalities, even though in many other countries, RRs are higher. The reason is that, with an average under-5 mortality level of 303/1,000, the RR in Niger cannot be very high. The same is true for low RDs at very high and very low overall rates. Bangladesh, with an overall professional delivery attendance level of only 8%, for example, exhibits a necessarily low RD in such care.

The observed general patterns in which the RR and RD are associated with the overall level of the outcome cannot be fully clarified by the mathematical ceilings. These ceilings only play a role at high overall rates (for RR) or very high and low overall rates (RD).

## Discussion

### Summary

Our paper shows that the magnitude of both relative and absolute socio-economic inequalities in health-related outcomes is empirically related to the overall level of these outcomes. Relative inequalities, using the Rate Ratio as measure, tend to be larger at lower overall levels (e.g. of mortality). Absolute inequalities, using the Rate Difference as measure, tend to be low at both very low and very high overall levels. Our paper demonstrates that the magnitude of the RR and the RD is bound by mathematical ceilings. These ceilings partly explain the empirical patterns described above. Low RRs at very high overall levels, for instance, are a necessity, not an accomplishment. They reflect the fact that rates in all wealth layers need to be very high in order to uphold a very high overall level. Yet, even where mathematically-defined ceilings do not play a role, the magnitude of absolute and relative inequalities is correlated with the overall level.

Rising RRs with declining overall levels are, however, not a necessity. There are countries with low mortality rates *and *low RRs. In Uzbekistan and Kazakhstan, for example, the RR in under-5 mortality is low (RR = 1.15 and 1.01 respectively), despite the comparatively low overall under-5 mortality levels in these countries (55/1,000 and 48/1,000 respectively). Similarly, the RD showed variation around the trend-line at most overall levels. Moreover, the exact empirical patterns varied between the specific health-related outcomes, showing that the relationship between relative and absolute inequalities on the one hand, and overall levels on the other, is not as rigid as sometimes suggested.

### Evaluation of methodology

Our results are based on DHS data, which uses standardized core questionnaires that generally allow for comparisons across countries. Although there is some uncertainty around the precise estimates for individual countries, it seems unlikely that this explains the systematic patterns observed. As DHS comprises a broad set of countries (representing various regions, and political, economic and cultural contexts), we expect that the patterns described are not dependent on the selection of countries for which DHS data are available. Also, we examined a broad set of outcomes. We expect an approximately similar range of patterns for other outcomes that are associated with socio-economic status.

A wealth index, based on household ownership of assets, was the socio-economic characteristic used in this study. When using maternal education, we found similar patterns (results available upon request).

Our empirical findings are based on a cross-sectional cross-national analysis, and are therefore directly relevant for international comparative studies. Patterns across countries in one period of time can, however, not necessarily be interpreted as also reflecting changes over time within countries. There are, however, indications that the observed tendencies of the RR and RD are also seen over time. In Western Europe, declines in total mortality among adults between the 1980s and 1990s were accompanied by increasing relative inequalities in mortality between socio-economic groups [2,14,15]. In developing countries, there is evidence that the decline in childhood mortality between the 1970s and the 1990s was accompanied by declining absolute socio-economic mortality inequalities, and stable or widening relative inequalities [16,17].

Our findings are important, not only for international comparisons of low and middle-income countries, but for all studies in which (health-related) inequalities are compared between populations. When comparing mortality inequalities between European countries [18], for example, or when monitoring time-trends in inequality [7], differences in overall mortality levels need to be taken into account. Also when comparing health inequalities between age groups it is important to take into account the fact that overall mortality rises with age. Indeed, relative inequalities tend to decline with age, while absolute differences increase dramatically [19-21].

We used the most simple measures of relative and absolute inequality (the Rate Ratio and the Rate Difference) to illustrate the general tendencies and mathematical ceilings. Our findings can most likely be generalised to more sophisticated measures of relative and absolute inequality, such as the Relative Index of Inequality [4], the Slope Index of Inequality (SII) [4], and the Generalized Concentration Index [5]. The mathematical ceilings to the Concentration Index have been described elsewhere [22]. In a previous study we have reported a similar relationship between the SII and the overall level to the one reported here for the RD [23].

### Explaining the patterns

As mentioned above, the observed general patterns in which the RR and RD are associated with the overall level of the outcome cannot be fully clarified by the mathematical ceilings. Further interpretation and appraisal of the observed patterns can be enhanced by placing them in an explanatory framework. An example of such a framework is the diffusion of innovations theory [24]. According to this theory, innovations tend to reach the better-off first before trickling down to the lower classes. This would lead to high relative inequalities at the early phase of the diffusion process, and to a decline later onwards [8]. Differential diffusion of innovations has been observed for a number of phenomena, such as the smoking epidemic in developed countries and the obesity epidemic [25,26]. Indeed, the observed pattern of high relative inequalities at low levels of health care use and the low inequalities at high levels of health care use is conform expectations based on the diffusion of innovations theory.

### Implications for monitoring health inequalities

Our study shows that not only the RR [7], and not only the RD [8], but both are associated with the overall level of the outcome. Preference for either measure can therefore not be based on (supposed lack of) these general tendencies.

At the same time these tendencies are not necessities. Scanlan argues that increasing RRs are nearly inevitable as mortality rates decline [7]. Positive examples, however, demonstrate that keeping relative inequalities low when mortality levels decline, is attainable. This is important, both for policy makers and researchers, especially those who assume that rising inequalities with declining mortality levels are inevitable. Also the RD varies around the trend-line at most overall levels. This implies that both the RR and the RD are not entirely determined by overall levels and that both can be meaningful measures for monitoring inequality.

Conversely, small RRs at high overall levels are almost inevitable, as are low RDs at very low and very high overall rates. Ultimately, very low mortality levels are only attainable when absolute mortality inequalities are low. This should be taken into account when monitoring inequalities. The RR and the RD are therefore only useful for monitoring when the relationship of these measures with the overall level of the outcome is taken into account. Also when setting targets for reducing health inequalities, e.g. a 25% reduction in health inequalities in Europe [3], it is important to take into account the context in terms of overall rates, and to carefully consider the measure used for monitoring progress.

Whereas there are no standard recipes, we will give some suggestions on how the overall level of the outcome can be taken into account when monitoring inequalities.

When populations with similar overall levels of the outcome are compared, the RR and RD are both meaningful measures for monitoring. Malawi and Peru, for example, exhibit a similar overall level of professional delivery attendance (ca. 55%). Yet, Malawi is doing substantially better in terms of equity in the provision of such care (RD = 23) than Peru (RD = 60). When using the RR, one should, however, be aware that its magnitude can be highly sensitive to whether the outcome is defined positively or negatively, as we demonstrated for skilled delivery attendance. For certain outcomes (e.g. mortality), a negative definition is conventionally used, whereas for others (e.g. immunisation) a positive definition is more common. We warn against uncritical use of common but arbitrary definitions of health-related outcomes in either positive or negative terms. Each definition describes another aspect of the empirical reality, and it can be meaningful to describe inequalities according to both.

When populations with different overall levels are compared, one can assess whether the population with smallest inequalities theoretically could, given its corresponding mathematically-defined ceiling, have reached the higher inequality observed in the population with which it is compared. If the magnitude of inequality of one of the populations seems to be restricted by the mathematically-defined ceilings, such direct comparisons may not be very meaningful. For example, whereas the RD in professional delivery care in Bangladesh (RD = 17) is lower than in India (RD = 48), a direct comparison between the two on basis of the RD may not be very meaningful as absolute inequalities in Bangladesh are necessarily low given its overall level of delivery care (8% vs. 34% in India).

A solution to both of the above issues would be to use Odds Ratio-based measures of inequality. These measures are not bound by mathematically-defined ceilings, and they are insensitive to whether the outcome is defined positively or negatively. While these are obvious advantages of the Odds Ratio, it has the disadvantage that it is hard to interpret by non-researchers [27], who may tend to misinterpret this measure as a RR [28]. Moreover, while the insensitivity of the OR to positive or negative health outcomes makes it immune to arbitrary decisions on outcome measures, it does not stimulate the researcher to be explicit in choosing for either a positive or a negative outcome indicator. An explicit choice is valuable in cases where positive and negative indicators, such the immunisation rate versus the non-immunisation rate have different policy implications.

International patterns, as presented in this paper, can also be used for monitoring. A country's performance in terms of health inequality can be assessed with reference to other countries with similar overall levels of the outcome. The trend-line, representing the average performance of countries at a given overall level, can be used as benchmark. For example, Malawi, with an overall level of professional delivery attendance of 55% and an RD of 23, is doing well compared to the trend-line presented in Figure 2c. Alternatively, the best possible attainment at a given overall level, or a predefined target may be used as reference. Finally, expectations based on the diffusion of innovations theory, can be used as framework for evaluating observed inequalities.

It can be useful to assess group-specific rates in addition to summary measures of inequality, for example when monitoring differential diffusion of innovations through a population. Again, it is important to take the overall level of the outcome into account. If not, group specific rates may become an indicator of the overall performance of a country, rather than being an indicator of its distribution. Group-specific rates can be benchmarked similarly as described above, using international comparisons.

Summarizing, both absolute and relative inequality measures can be meaningful for monitoring socio-economic health inequalities, provided that differences or changes in the overall level of the outcome are carefully taken into account. Our paper gives advice on how to take this overall level into account when monitoring these inequalities and presents data that can be used for benchmarking of low- and middle-income countries in the field of maternal and child health.
